# Stigma on epileptic patients attending the outpatient clinic of Soba University Hospital and the National Center for Neurological Science (NCNS) Khartoum, Sudan

**DOI:** 10.11604/pamj.2019.32.93.17511

**Published:** 2019-02-27

**Authors:** Muwada Bashir Awad Bashir, Shahd Mohammed Abdalla, Ngwayu Claude Nkfusai, Frankline Sanyuy Nsai, Rosaline Yumumkah Cumber, Joyce Mahlako Tsoka-Gwegweni, Samuel Nambile Cumber

**Affiliations:** 1 Discipline of Medicine and Surgery, Faculty of Medicine University of Khartoum, Kkartoun, Sudan; 2 Section for Epidemiology and Social Medicine, Department of Public Health, Institute of Medicine, The Sahlgrenska Academy at University of Gothenburg, Gothenburg, Sweden; 3 Department of Microbiology and Parasitology, Faculty of Science, University of Buea, Buea, Cameroon; 4 HIV Free Project, Center Region, Cameroon Baptist Convention with Funds from PEPFAR through CDC Atlanta, USA; 5 Department of Public Health, Faculty of Health Sciences, University of Buea, Buea, Cameroon; 6 Department of Political Science, University of KwaZulu-Natal, Durban, South Africa; 7 School of Nursing & Public Health, College of Health Sciences, University of KwaZulu-Natal Durban, South Africa; 8 Faculty of Health Sciences, University of the Free State, Bloemfontein, South Africa

**Keywords:** Stigma, types, epilepsy, patients, Sudan

## Abstract

**Introduction:**

Epilepsy is one of the most prevalent neurological conditions for people of different age group, race, gender and socio-economic status in various nations, affecting 50 million people around the world; 80% of them living in developing countries. In Sudan, literature has well documented epilepsy stigma and it related elements of labelling, negative typecasting, isolation, devaluing and discrimination that are significantly affecting patients living standards and social life. This study focuses on understanding the prevailing, different types of stigma among people living with epilepsy (PWE) in Sudan and to determine its frequency in connection with demographic factors and patients coping ability of PWE.

**Methods:**

A health facility-based cross-sectional descriptive study was conducted on 270 people between the ages of 16-70years who are affected by epilepsy in an outpatient clinic. Standardized questionnaires were used to interview the patients with assistance gotten from their relative where necessary. The data is analyzed using SPSS version 20. The study was conducted in the outpatient clinic of The National Center for Neurological Sciences (NCNS), Ibrahim-Malik Teaching Hospital Sudan.

**Results:**

The study realized that 16.6% of the patient had a positive self-stigma score, half of them having coaching stigma and 25% courtesy stigma. One third of people with epilepsy had poor coping score that was strongly determined by self- stigma and courtesy stigma score.

**Conclusion:**

Parents and relatives related sigma is a major problem facing people of epilepsy in Sudan; thus, raising such group awareness and education about the disease can markedly improve the quality of life of people with epilepsy in Sudan.

## Introduction

Epilepsy is a disease characterized by an enduring predisposition to generate epileptic seizures and by the neurobiological, cognitive, psychological, and social consequences of this condition. [1]. Epilepsy is also, one of the most prevalent neurological conditions affecting people of different age groups, race, gender, and socioeconomic status; thus, indicating that no one is immune to epilepsy [1]. An estimate of 50 million people have been affected by epilepsy around the world, with 80% of them living in low and middle-income countries [2]. Epilepsy account for 0.5% of the global burden of disease and has serious implications in term of healthcare needs [3]. Despite being a common neurological disorder, it seems to be way behind in terms of understanding in low and middle-income countries due to traditional, socio-cultural and religious believes which attributes the disease to demonic possession and evil spirits and misconceptions about disease transmissibility [3]. According to Coffman (1963) who introduced the stigma concept that is still being used, “Stigma can be seen as a relationship between attribute and stereotype and is a reference to depreciative attributes, weakness or disadvantages.” In other words, stigmatized individuals possess some characteristics which are different from others [4].

The stigma of epilepsy and the social burden remain the main challenges faced by those suffering from epilepsy around the world, and not the signs itself [5]. Aside from the great worry of having seizures in an inappropriate situation. stigma has great influence on patient's adherence to treatment [5]. However, in attempts of understanding stigma experience of people with epilepsy (PWE), different concepts that highlight several types of stigma have emerged: felt stigma, enacted stigma, stigma coaching and courtesy are all variant characterizations of both personal and social manifestations of stigma perception by PWE. Felt or self-stigma refers to the patients' self-feeling of shame and fear of oppression. Enacted stigma, however, is the actual act of discrimination against PWE based on their medical status that is practiced by their surroundings. Stigma coaching is a notion that refers to the behavior of parents ashamed of their children's disease status who, as result, teach their children that epilepsy is an undesired or burden that they all must tolerate. Parents' manner of approaching the disease in forms of training given to children by their parents, emphasizes their inner feeling of disability and exposes them more to stigma. Lastly, stigma courtesy describes the overriding impact of stigmatizing PWE that extends to affect their families, relatives and close social circles [5]. Therefore, In this study, we aim to study the different forms of stigma prevailing among a sample of Sudanese people with epilepsy (PWE) attending the National Center for Neurological Sciences (NCNS), Ibrahim-Malik Teaching Hospital Sudan and to determine its frequency as well as possible association with demographical factors and patients coping ability.

## Methods

### Study design and setting

The study is a cross-sectional study that was conducted in the outpatient clinic of The National Center for Neurological Sciences (NCNS), Ibrahim-Malik Teaching Hospital, established in October 1977. The center located in Al Sahafa Shariq town, having over 270 patient's bed, offers different medical specialties' services for a population of over 100,000 people. The center is also affiliated to the University of Khartoum under the umbrella of Sudan Board for Medical Specialization and Federal Ministry of Health as training facility for medical residents and house offices. The outpatient clinic of Soba University hospital -the teaching hospital of Khartoum University- was established in 1975 as the main training hub for the medical students, Post-graduate students, nursing college students, medical laboratory Sciences students and other allied health professions students affiliated to University of Khartoum. It is a tertiary hospital offering different medical specialties and services, recognized by Sudan Board for Medical Specialization and Federal Ministry of Health for the training of registrars and house offices.

### Study population

**Inclusion criteria:** People with clinically confirmed diagnosis with epilepsy according to the definition of the international league against epilepsy (A disease characterized by an enduring predisposition to generate epileptic seizures, and by the neurobiological, cognitive, psychological, and social consequences of it) who are aged from 16 to 70 years old.

**Exclusion criteria:** Participants who were below 16 years, owing to difficulty in apprehending and clearly recalling coaching and courtesy stigma by participants of this age [6] and participants who are above 70 years because of the high likelihood of recalling bias, due to senile memory and cognition impairment [5].

**Sample size and sampling:** A convenient sampling frame was used. It consisted of a total coverage of all individuals with epilepsy attending the clinic from 25th November till 25th of December 2017, using administered questionnaires

### Data collection and analysis

Interviews with patients were carried out using structured questionnaire composed of 21 both standardized and self-developed questions based on previously conducted similar studies [5, 7, 8]. Questionnaires were filled by trained nurses and research assistants. With respect to assessment of various types of stigma, self -stigma was measured using a standardized question of three points scale and attainment of 0 or 1 score indicates patients is free of stigma. The questions included asked about patient's perception of others avoidance or discomfort and their own feeling of inferiority because of their disease. They also included information about whether thy think that their communities believe that epilepsy is contagious and caused by demons and are acting accordingly towards them. Enacted stigma was assessed using questions that were developed by the author based on previous similar studies [5, 7, 8]. The questions addressed information about patients experience of discrimination in school, marital rejection and employment deprivation because of their disease, also how people react to patients' epileptic fits and their ability to disclose their sickness to others. To measure these, seven yes or no questions were used. As each answer with yes scores one, no answer scores 0.

A cutoff point of 3 and above was considered as positive enacted stigma case. Similarly, stigma courtesy and coaching were also assessed using self-developed 3 points yes or no questions, for which a score of 2 and above was considered positive. Courtesy were measured by asking about how parents react to their children affection by epilepsy and relatives feeling of sham, while coaching scope asked about weather patients hear from their parents' negative statement that assert their disability status as PWE. Before commencement of the study, the questionnaire was tested upon 14 cases that demonstrated well understanding and response to all the questions and were later excluded from the study participants. Data were collected in master sheet. Relatives of the epileptic patients were occasionally asked when there is need for clarifying information. Coping conditions were measured by three questions about patients thinking of their disease condition as a negative bear; feeling of stress due to being diseased and reliance on secrecy to mask and alleviate the negative consequences of the disease. Each of the three questions had a score of 1 and patients who attained a score of 2 or more were considered as of poor coping status [5].

Descriptive data including patients' demographic characteristics and frequencies of different types of stigma were presented as numbers and percentage, while conclusion about significant associations between patients' demographic characteristics, disease severity, levels of perceived stigma and coping levels were tested by using logistic regression analysis. Independent variables (socio-demographic characteristics, frequency of seizures and the type of stigma of interest) were regressed against dependent ones (self-stigma, enacted stigma, parents related stigma and coping score). Our reference outcomes for the regression analysis were male for gender; no education for education level; once per month for seizures frequency as the least frequency; age group 16-20 years for age and negative score achievers for each type of stigma and coping ability, taking p<0.05 as limit of significance and using SPSS program version 20.

**Ethical consideration:** Ethical clearance was obtained from the department of Community Medicine in University of Khartoum and Khartoum ministry of health. Permissions from NCNS and Soba University Hospital were also obtained to conduct the study in the outpatient clinic. Research purposes and objectives were explained to participant in clear simple words and informed written consents were obtained from them. They were also notified about their rights to withdraw at any time without any deprivation. Participant's information was handled with high confidentiality only by the study researchers.

## Results

The study enrolled 270 participants (n=270) aged 16-70 years. 55.6% (148) were males and 44.4% (122) were females. 69.4% (187) aged 31years and above, while 30.5% were below 31years. 41.6 % of the participants were at primary level of education. Regarding disease severity, 28% of them reported having 1-3 seizures per month, while 14% had more than one seizure per week. Almost all the participants were on medications (Table 1).

**Table 1:** Socio-demographic data

Variable	Frequency	Percent
**Gender**		
Male	148	55.5%
Female	122	44.4%
**Age**		
21-30 years	82	30.5%
31-40years	90	33.3%
Above 40 years	97	36.1%
**Level of education**		
Non-educated	30	11.0%
Primary	112	41.6%
Secondary	75	27.7%
University and higher education	52	19.3%
**Total**	270	100%

**Self-stigma:** Sixteen percent of the study participants had positive score of self-stigma. Neither gender or age were of significant association with self-stigma score. However, the probability of attaining positive self-stigma score was high among lower education levels (p-value 0.03) and those with low coping ability score ( p value: 0.001), while higher among those who score positive enacted (p-value <0.000) and coaching stigma (p-value 0.028) score (Table 2).

**Table 2:** Felt stigma and education levels, enacted stigma and coaching stigma

Felt stigma score	Self-Stigma Positive	Self –stigma Negative
Education level	P value =0.03 < 0.05	
Non-educated	14	16
Primary	12	91
Secondary	10	65
University and higher education	7	45
**Enacted stigma**	**P value = 0.000 < 0.05**	
Positive	42	26
Negative	1	190
**Coaching stigma**	**P value = 0.028 < 0.05**	
Positive	35	26
Negative	8	63
**Total**	43	206

**Enacted stigma:** Nineteen percent of the study participants reported that their communities believe that epilepsy is contagious, while 36.8% said they attributed it to demon possession. 38.8% of the participants mentioned that people feared approaching them when they had seizures in public; hence, they have not been receiving help from spectators during fits episodes. Regarding informing their communities about their disease conditions, 8% reported that it was against their will compared to 77% who voluntarily apprised their medical condition with others. Yet, 15% are hiding it not preferring to publicize their disease status.

Only five percent of our PWE reported experiencing prejudicing behaviors form their teachers in schools, while the rest of the participants either weren't diseased by schooling age or abandoned education because of their medical condition. Also, 8.3% conveyed at least one incident of losing an employment chance due to having epilepsy, 47.2% never tried to get a job, and 11.1% lost at least one marriage proposal owing to their medical conditions. Generally saying, twenty-five percent of PWE acquired positive enacted stigma score that was, furthermore, positively associated with disease severity measured as frequency of seizures (p value: 0.001) and patients self-stigma score (P value: 0.045) (Table 3).

**Table 3:** Enacted stigma and self-stigma and enacted stigma and disease severity as frequency of seizures

Self - stigma		Enacted stigma positive	Enacted stigma negative
Self-stigma		P value 0.045	
Positive	43	35	66
Negative	206	29	136
**Frequency of sizers**		**P value 0.001**	
1 per month		14	155
1 per week		25	40
More than one per week	30	29	7
**Total**		68	202

**Coaching stigma:** Positive coaching stigma score was counted among 59% of the study participants. No significant association was demonstrated between coaching stigma and any of the participants' socio-demographic variables or other types of stigma.

**Parents/Relatives (courtesy) stigma:** Twenty five percent (67) of the participants had at least once experienced stigmatization from their relatives. Courtesy stigma was found to be positively affecting coaching stigma (p-value: 0.029), while negatively influencing coping ability (p-value: 0.03) (Table 4).

**Table 4:** Coping ability and courtesy stigma and coping ability and self-stigma

		coping ability low score	coping ability high score
Courtesy stigma		P value =0.03	
Positive	67	59	8
Negative	201	22	119
**Self - stigma**		**P value = 0.000**	
Positive	43	38	3
Negative	206	35	173
**Total**		81	180

**Coping Assessment and psychological counseling:** Fifty-two percent of PWE considered their epilepsy as a moral weight that they could not live with, while 16.7% were always self-stressed about their condition and 46.1% prefer to hide their medical conditions. Only 8% of PWE received psychological counseling about their condition. Overall coping score was low among 30% of PWE and 40% of those who were stigmatized by their parents and relatives had poor coping score. It was also found to be significantly lower among participants who scored high in self-stigma (p value: 0.001) and courtesy stigma (p value: 0.03) (Table 4).

## Discussion

The aim of this study was to investigate the burden of different forms of stigma among PWE and their possible associated factors with respect to patient's socio-demographic characteristics, treatment compliance and other types of stigmatization. For the purpose of discussion, each type of stigma will be addressed separately for review, comparison, and relevant clinical implications.

Considering self-sigma, the study showed that 16.6% of the patients reported having positive self-stigma. This percentage, though is low compared to figures reported in studies from developed communities like Netherlands (30%), Poland (56%) and France (62%) [8], is similar to results from other studies [9, 10], including one from Sudan [11]. The noticeable low prevalent of self-stigmatization feeling among Sudanese communities suggested the possible role of religious and social ties in alleviating patients' sufferance. Another important factor is the way in which low socio-economic standards of living creates a low scale criterion of self-image and personal expectations which, in turn, influences patient's acceptance of their disease status and helps to relieve their stigmatization feeling [5].

In this study, self-stigma scores were also found to be negatively associated with educational level as highly educated patients were of less probability to perceive self-stigma. Such findings are inline with stated conclusion from similar studies [8-10]. Whereas self-stigmatization is most likely to be associated with wrong beliefs about disease and its linkage to devil possessions, these attributes are less expected to prevail among patients who are highly educated. In addition, selection bias, in such context, is another possible justification for our findings. PWE who were severely incapacitated by their diseases that their educational progress was hindered are more liable to apprehend stigmatization and perceive it. Although our study failed to capture the same significant effect among the highest education group, because of probably the low sample size with and small presentation of the very highly educated groups, it is well reported that epilepsy has accumulation effect of depriving patients in term of social wellbeing and further medical care and treatment [6].

To investigate whether patient's self-perception of stigma is shaped by their encounter of discriminative acts in their surroundings, we measured possible association between self -stigma and enacted and parents/relatives related stigma. The finding that enacted-stigma is positively related to self-stigma is acceptable in the logic: patient's self-perception of loneliness because of their disease is intensified by what they face in their surrounding that assert their feeling of being disabled. However, this statement conceptually interferes with definition of self-stigma as the feeling internally generated and apprehended by patients [5].

When it comes to gender, our study didn't find any significant association of it with felt stigma. In line with previously reported findings of studies from Sudan [4, 5]. The statistical power and small sample size in our study is properly masking the relation path, despite the equal representation of the two genders in our study, as females high susceptibility to stigmatization due to mental illnesses including epilepsy is well documented through several studies from developing countries that mimic Sudan socio-cultural fabric [10].

Approximately one third of the study participants mentioned that their communities believe that epilepsy is caused by Demon possession, while for contagiousness; one fifth thought that their disease is dealt with as contagious. This figure, which are higher than the reported in [10], symbolizes the negative discriminatory attitude, disease fear and devaluing experienced by PWE, escalating their perception of stigma. Again, almost 77% of PWE disclosed their disease status with their communities; in consistent with the figures reported in [10], indicating that, for majority of the patients, sharing information about their disease with their surrounding was not difficult. The religious component with respect to the Sudanese society understanding of epilepsy is a two-sided sward. While disease attribution to demon possession disserve patients experience, mental illness consideration as divine selection of blessed one's results in better acceptance of their disease by their communities. More even, these figures are self-explained by the reported low figures of enacted stigma score.

Regarding discrimination acts by teachers, employability and marital proposals, the retrieved findings of 5%, 8% and 11%, respectively, are relatively lower when compared to similar study from Sudan [5]. Being a direct indicator of enacted stigma by community, these low figures are explainable by, other than the small sample size, the selection inclusion of patients who are of good medical follow up and treatment regime which further qualified them for better functionalism as patients. A significant difference in the average frequency of seizures was found between those who were affected and non-affected by enacted stigma. Higher frequencies of seizures predicted higher probability of experiencing enacted stigma. This indicates that patients' ability control disease manifestations which triggers community disabling acts is lost with sever disease with frequent epileptic fits.

One of each two PWE had perceived coaching stigma, while one of each four patients had courtesy stigma. This goes in direction with findings from [10]. Actuated by care and wariness parents tend to over direct their diseases children to avoid accidents or situations that might endanger them in a manner that emphasis their disability for them [10]. Another causes for such behavior could be their own perception of inferiority and shame of their children diseases especially within the relative and family context because those (relatives), in turn, are posing an oral burden on them. This fact is also supported by our finding that courtesy stigma positively affect coaching stigma; The higher the perception of relatives of embarrassment and shame the higher the parents attitude towards coaching their children to disability because of their diseases.

Almost half of PWE prefer to conceal their condition and similar count are stressed about because of their medical conditions resulting in an over one third of the participants having poor coping ability with their disease. This figure is comparatively higher than the reported in other studied of similar approach [5, 8]. Patients ability to adjust to their disease status is logically related to their comfort with it. The thing that patient's high stated moral burden and stress is not indicative for. The findings that coping ability is negatively affected by self-stigma and coaching-stigma but not enacted stigma score is in partial coherence with findings from [10] a similar study from Jordan [11], indicating that patients self-adjustment capacity is determined by the amount of devaluing they receive from their close everyday surrounding rather than strangers reactions. The things which further suggest the involvement of cumulative time exposure factor in such skills attainment.

## Conclusion

Coaching and curtesy stigma are the mostly abundant forms of stigma among PWE in Sudan, followed by enacted stigma and then self-stigma, which is the least frequent. The higher the educational level of the patients the lower their perception of self-stigma, while enacted and coaching stigma acts reversely by positively affecting self-stigma. Out of each four patients, one encounters enacted stigma. This is more likely when disease is more sever and patients' experiences seizure very frequently. Parents attitude of training their children to be stigmatized, emphasizing their disability, is the number one problem faced by PWE. Such parents attitude is likely imposed by relative's stigma as how much they and family surroundings are ashamed of the diseased one. As one third of PWE are of low adjustment scores, patient's ability to cope with their disease is directly affected by their level of perception of self and courtesy stigma; hence, patients, parents and close relatives education about the disease would improve patients coping capacity.

### What is known about this topic

- The burden of epilepsy may be due to physical hazards of epilepsy resulting from unpredictability of seizures; the social exclusion resulting from the negative attitudes of others towards PWE;
- Patients with epilepsy are one of the most vulnerable in any society. This vulnerability may be partly attributed to the disorder itself or it is related socio-cultural projections;
- Stigmatization leads to discrimination, and PWE have been the target of prejudicial behavior in many spheres of life, over many centuries and in many cultures.

### What this study adds

- Close social surroundings (Parents and relatives) attitudes are the major burdening form of stigma and disabling acts for PWE in Sudan in comparison to enacted and self-stigma;
- Educating both patients and their social circles behaviorally about the disease along with the importance of controlling seizures with medications and triggers avoidance could markedly minimize the amount of stigma encountered by the patients;
- Patient's ability to cope with their diseases is more likely to be determined by their closed circles (family and relatives) behaviors rather than the general community acts towards their disease.

## Competing interests

The authors declare no competing interests.
